# Allergology 2030 – 75 years of the DGAKI: Early detection, more effective prevention, better treatment: DGAKI Position Paper on Preventive, Evidence-Based, Precision-Oriented, and Integrated Allergology in Germany

**DOI:** 10.5414/ALX02657E

**Published:** 2026-10-22

**Authors:** Eckard Hamelmann, Marek Lommatzsch, Wolfgang Pfützner, Martin Wagenmann, Bettina Wedi, Margitta Worm

**Affiliations:** 1 Department for Pediatrics and Adolescent Medicine, Children’s Center Bethel, University Hospital OWL, Bielefeld University, Bielefeld,; 2 Clinic and Polyclinic for Internal Medicine, Department of Pulmonology, Rostock University Medical Center, Rostock,; 3 Department of Dermatology and Allergology, Division of Allergology, Marburg University Hospital, Philipps University of Marburg, Marburg,; 4 Department of Otolaryngology, University Hospital Düsseldorf, Heinrich Heine University Düsseldorf, Düsseldorf,; 5 Department of Dermatology, Allergology, and Venereology, Hannover Medical School (MHH), Hanover, and; 6 Department of Dermatology, Allergology, and Venereology, Division of Allergology and Immunology, Charité – University Medicine Berlin, Charité Mitte Campus, Berlin, Germany

**Keywords:** allergy, allergology, prevention, precision medicine, healthcare, National Action Plan, DGAKI

## Abstract

Allergic diseases are among the most common chronic conditions in Germany and may affect patients throughout the life course, often with multimorbidity and, in some cases, life-threatening manifestations. Despite substantial advances in immunology, molecular diagnostics, allergen immunotherapy and targeted therapies, allergology in Germany faces structural deficits in education and training, academic infrastructure, regional access to specialized care, interdisciplinary coordination, and the implementation of scientific innovation into routine care. This position paper of the German Society for Allergology and Clinical Immunology (DGAKI), published on the occasion of its 75^th^ anniversary, outlines a vision for “Allergology 2030”. Allergology should evolve from a predominantly reactive discipline towards a more preventive, evidence-based, precise and coordinated approach. This requires stronger academic structures, reliably secured allergological expertise, tiered and integrated care pathways, and robust infrastructures for translation, data and implementation. Because these challenges extend beyond individual specialties, institutions and sectors of care, the DGAKI calls for the development of a National Action Plan for Allergology in Germany. Together with relevant partners and stakeholders, such a plan should translate scientific evidence and identified unmet needs into shared priorities, measurable objectives, implementation mechanisms and evaluation. The overarching goal is to create the conditions for allergic diseases to be detected earlier, prevented more effectively and treated better.

## 1. Executive summary 

Allergic diseases are among the most common chronic conditions in Germany. They often begin early in life, can affect people throughout their entire lifespan, and frequently occur in conjunction with other conditions. They range from common chronic conditions such as allergic rhinitis, bronchial asthma, and atopic dermatitis to severe and potentially life-threatening reactions such as anaphylaxis. Allergies are therefore not minor conditions but major public health conditions with substantial individual, medical, and societal relevance. 

This disease burden is compounded by structural deficiencies. Allergy expertise is not sufficiently mandated in undergraduate, continuing, and advanced education; university and research infrastructure is limited; access to specialized care varies by region; and the management of severe, complex, and multimorbid conditions is often not sufficiently coordinated. At the same time, inadequate structural and financial conditions hinder the implementation of guideline-based diagnosis and treatment, as well as the transfer of scientific innovations into clinical practice. 

Yet modern allergology opens up new possibilities. Advances in immunology, molecular biology, and precision medicine enable increasingly sophisticated diagnostics and targeted therapy. Prevention and early detection are gaining in importance, as are the coordinated care of complex diseases and the translation of new findings into clinical practice. Allergology must therefore evolve from a predominantly reactive discipline toward a more preventive, evidence-based, precision-oriented, and coordinated approach to medicine. 

To achieve this, Germany needs strong university-based structures, reliably secured expertise in allergology, a tiered and networked care system, and efficient structures for translation, data management, and implementation. These tasks extend beyond the purview of individual specialties, institutions, and levels of care. The German Society for Allergology and Clinical Immunology (DGAKI) therefore calls for the development of a National Action Plan for Allergology in Germany and intends to shape its development scientifically in collaboration with its partners. The goal is to create the conditions necessary to detect allergic diseases earlier, prevent them more effectively, and treat them better in the future. 

## 2. Allergic diseases – The underestimated public health problem 

Allergic diseases are among the most common chronic conditions in Germany. Approximately one-quarter of the population is affected by an allergic disease; a significantly larger proportion tests positive for sensitization to potentially allergenic environmental substances. Allergies are thus not diseases affecting small groups of patients but widespread conditions with high individual, medical, and societal relevance [1, 2]. 

### Allergies often begin early in life – but affect the entire lifespan 

The burden of disease becomes apparent as early as childhood and adolescence. Germany-wide surveys show consistently high prevalence rates for bronchial asthma and allergic rhinitis. In KiGGS Wave 2 (2014 – 2017), a 12-month prevalence of 8.8% was determined for allergic rhinitis and 3.5% for bronchial asthma; this corresponds to more than one million and approximately half a million affected children and adolescents in Germany, respectively. For atopic dermatitis, a lifetime prevalence of 14.3% was reported in KiGGS Wave 1. Allergic sensitization is also common as early as childhood and adolescence [3, 4]. 

The early onset of these conditions is also relevant because allergic diseases often do not occur in isolation. Atopic dermatitis, food allergy, allergic rhinitis, and asthma may follow one another or coexist throughout the course of an individual’s medical history. Allergic diseases are therefore often chronic and multimorbid conditions whose manifestations affect various organs and can accompany patients over long periods of their lives [5]. 

However, allergies are by no means exclusively diseases of young people. Their prevalence remains high even in adulthood. Nationwide representative data show that just under one-third of adults will have at least one medically diagnosed allergic disease at some point in their lives. Allergic diseases are observed well into old age and can also develop for the first time at that stage. Allergology is therefore a field that spans the entire lifespan [1, 6]. 

### From the chronic burden of disease to life-threatening reactions 

In addition to their prevalence, the wide range of disease severity is of particular significance. Allergic rhinitis, bronchial asthma, and atopic dermatitis can persist for years or decades and significantly impair quality of life, sleep, performance, school, education, and career [6]. 

In addition, allergic diseases can present with acute, severe, or life-threatening courses. Anaphylaxis represents the most severe manifestation of an immediate-type allergic reaction; its most common triggers include foods, insect venoms, and medications. Patients with severe forms of the disease or multiple concurrent atopic conditions, in particular, require coordinated, multidisciplinary, and long-term care [7]. 

Allergology thus encompasses a spectrum ranging from very common chronic conditions to potentially life-threatening reactions. This spectrum contradicts the still widespread perception of allergic diseases as predominantly seasonal or minor conditions. 

### High burden of disease – high societal relevance 

The health consequences are compounded by significant societal burdens. Chronic symptoms, reduced functional capacity, and absences from school and work lead not only to direct treatment costs but also to substantial indirect and economic costs. Due to their high prevalence, often early onset, and in some cases lifelong course, the burden of allergic diseases thus extends far beyond the individual diagnosis [8]. 

Allergic diseases are common chronic conditions that are often multimorbid and, in some cases, life-threatening. However, their high medical and societal significance is countered by a structural framework within the German healthcare system that has so far failed to adequately address this disease burden. 

## 3. Structural deficits in allergology in Germany 

The high burden of allergic diseases in Germany is met with healthcare, research, and training structures that do not adequately address their medical and societal significance. Allergology is an interdisciplinary field that bridges, among other fields, dermatology, pediatrics, pulmonology, otolaryngology, and other clinical and scientific disciplines. It is precisely this interdisciplinary nature that is one of its strengths – yet without binding structures, it can also lead to fragmented responsibility. 

In Germany, allergology is not recognized as an independent medical specialty but is organized as an additional postgraduate qualification (“Zusatz-Weiterbildung”) available to physicians from several specialties [9, 10]. 

### 3.1 Medical education, postgraduate training, and continuing professional development: Allergy expertise is not sufficiently embedded in the educational structure 

Allergy-related content is primarily taught within various medical specialties during medical school; it is not listed as a separate focus area for allergy in the National Competency-Based Catalog of Learning Objectives for Medicine (NKLM) [11]. 

Structural challenges also exist in postgraduate medical training. With the competency-based training regulations, the previously mandatory minimum training period for the subspecialty training in allergology has been eliminated. Consequently, ensuring a uniform scope of practical allergology training may become even more important. At the same time, sound continuing education depends on institutions that actually offer a sufficient range of allergic diseases, diagnostic procedures, and therapies [12, 13, 14]. 

Since expertise in allergology cannot be acquired exclusively within a single specialty, organ-specific expertise and interdisciplinary knowledge of allergology must be integrated. A lack of suitable training centers and structured continuing education opportunities poses a long-term threat to the next generation of qualified allergists and, consequently, to future patient care. 

### 3.2 Science and translation: High innovation momentum meets weak academic structures 

Allergology has evolved into a dynamic field of translational medicine. Advances in the understanding of immunological mechanisms, as well as new diagnostic and therapeutic options, are fundamentally transforming prevention, diagnosis, and treatment. 

This scientific dynamism is counterbalanced in Germany by limited independent academic structures. Allergy research, teaching, and specialized care are predominantly anchored in various clinical specialties. Individual newly created structures represent important developments but cannot replace a broader university-based foundation for allergology [9, 14]. 

The lack of permanent academic structures hinders the establishment of long-term research programs, the development of reliable career prospects for early-career researchers, and the continuous integration of basic science, clinical research, and patient care. This creates the risk of losing scientific potential and failing to translate innovations into clinical practice as quickly as possible. 

### 3.3 Care: Between local primary care and highly specialized allergy care 

The vast majority of allergy care is provided on an outpatient basis and across various medical specialties. While this interdisciplinary approach is fundamentally a strength, it requires sufficient expertise in allergology, appropriate reimbursement structures, and effective networking. It is precisely here that significant shortcomings exist. Limited reimbursement options hinder time- and resource-intensive, guideline-based diagnosis and treatment; at the same time, access to qualified allergy care varies by region [14, 15]. 

These structural limitations are particularly evident in patients with severe, complex, or multimorbid allergic diseases. They often require the expertise of multiple specialties and access to specialized diagnostics and therapy. However, care is too rarely systematically integrated across outpatient, inpatient, and university-based medical settings [14]. 

The Network of Comprehensive Allergy Centers (CAC), initiated by the DGAKI in 2014 and supported by several medical societies, already provides a framework for the interdisciplinary care of complex allergic conditions. However, the number and regional availability of such centers are limited; at the same time, there is a lack of sustainable structural funding for the tasks associated with interdisciplinary care, networking, and translation [16]. 

Another example of the discrepancy between medical evidence and the reality of care is allergen immunotherapy (AIT). As a disease-modifying therapy, it holds a special place in allergology; however, its potential is not being fully realized in clinical practice [17, 18]. 

### 3.4 Prevention and early detection: Findings have not yet been sufficiently integrated into clinical practice 

With evidence-based recommendations for allergy prevention and increasing knowledge about risk factors and early disease manifestations, opportunities now exist to detect allergic diseases earlier and influence their progression. However, these opportunities have so far been integrated into existing healthcare structures only to a limited extent [19]. 

In particular, there is a lack of sufficiently established structures to identify risk constellations at an early stage, implement evidence-based preventive measures, and initiate qualified diagnosis and treatment in the event of early disease manifestations. The interfaces with pediatrics, primary care, and other areas of primary care are of particular importance in this regard. 

**The burden of disease and existing structures are out of balance**

The shortcomings described above reinforce one another:

- Weak academic structures hinder research, teaching, and support for early-career researchers
- Shortcomings in continuing education jeopardize future expertise in allergology; fragmented care pathways complicate the treatment of complex diseases.
- A lack of implementation structures delays the transfer of scientific findings into prevention and care.

The central challenge facing allergology in Germany therefore does not lie in a lack of scientific knowledge or medical options. Rather, there is a lack of sufficiently binding, interconnected, and sustainably supported structures to make existing knowledge and medical progress available to patients nationwide. 

Against this backdrop, the question arises for Germany: What medical and scientific capabilities does a sustainable allergology require – and what must allergology look like in 2030? 

## 4. Allergology in 2030 – Future medical and scientific challenges 

Allergology is at a turning point. Advances in immunology, molecular biology, and precision medicine are enabling an increasingly nuanced understanding of allergic diseases and opening up new possibilities for prevention, diagnosis, and treatment. At the same time, multimorbidity, demographic trends, and environmental and climatic factors are changing the demands placed on allergological medicine. 

The challenge in the coming years, therefore, is not merely to maintain existing care. Scientific progress must be harnessed to detect allergic diseases earlier and, where possible, prevent them; to make effective diagnostics and treatments available; to better treat severe and complex diseases; and to translate innovations into clinical practice more quickly. This gives rise to five key future challenges for allergology in Germany. 

### 4.1 Preventing and detecting allergic diseases earlier 

Many allergic diseases begin in the first years of life. For specific risk profiles and clinical presentations, evidence-based prevention strategies are available. At the same time, early manifestations of the disease can provide opportunities for timely diagnosis and intervention [19]. 

Allergology must therefore evolve from a predominantly reactive approach to a more preventive one. Evidence-based primary prevention measures must be consistently implemented, and new strategies for identifying at-risk groups and for early intervention must be scientifically evaluated. At the same time, it is essential to recognize early manifestations of allergic diseases in a timely manner and to initiate diagnosis and treatment in accordance with clinical guidelines [19]. 

This requires a life-course perspective: Prevention and early detection are particularly important in early childhood, but allergic diseases can emerge or change throughout the entire lifespan. The goal must be to consistently utilize existing prevention options and to influence disease development and progression at an early stage where scientific evidence supports such intervention [19]. 

### 4.2 Making evidence-based diagnostics and effective therapy accessible to all affected individuals 

Evidence-based diagnostic and therapeutic procedures are available for numerous allergic diseases. Nevertheless, not all patients benefit equally from these options. Differences in the availability of allergy expertise and coordination between the relevant medical specialties can lead to delayed diagnoses and the failure to initiate guideline-based therapies, or to their delayed initiation [14, 18]. 

A notable example is AIT. As a disease-modifying therapy, it can, in suitable patients, influence the course of the disease beyond mere symptom control. Its potential should be harnessed in accordance with the available evidence and guidelines. The same applies to established diagnostic procedures and innovative treatment options [17]. 

Allergology in 2030 must strive to ensure that access to quality-assured diagnostics and guideline-based therapy does not depend on place of residence, the primary treating specialty, or avoidable structural barriers. 

### 4.3 Coordinated care for severe, complex, and multimorbid allergic diseases 

Allergic diseases can affect different organ systems and occur simultaneously or over the course of a person’s life. Atopic dermatitis, allergic rhinitis, asthma, and food allergies may be interrelated. In addition, there are severe conditions such as anaphylaxis, as well as complex drug or insect venom allergies. The traditional approach of treating individual organ diseases therefore often fails to meet the needs of these patients [5, 7]. 

Severe, complex, and multimorbid conditions require care that transcends the boundaries of individual organ systems and medical specialties. Diagnostic and therapeutic decisions must be coordinated, and specialized expertise must be available when needed. 

New targeted therapies underscore this need. In particular, with type 2 inflammatory diseases, common immunological mechanisms may underlie various clinical manifestations. Treatment decisions should therefore increasingly take into account the entire spectrum of allergic and type 2-associated manifestations, as well as the individual disease burden [20]. 

The management of severe, complex, and multimorbid diseases is thus a central task of modern interdisciplinary allergology. 

### 4.4 Translating scientific advances into clinical care more rapidly 

Allergology is one of the most dynamic fields of innovation in medicine. Biomarkers, molecular diagnostics, new approaches to AIT, biologics, and other targeted therapies open up prospects for increasingly precise and personalized medicine [17, 20]. 

However, their benefits are realized only when scientific findings reach patients. For this to happen, the entire translational chain must function effectively: from basic research and clinical trials, through the evaluation of scientific evidence and guidelines, to their implementation in clinical care. At the same time, experiences and unresolved questions from clinical practice must feed back into research. 

Environmental and climate influences must be considered as a cross-cutting issue in this context. Changes in allergen exposure and other environmental factors can influence disease risks and disease progression and require the integration of clinical allergology with epidemiology, environmental medicine, and public health [21]. 

### 4.5 Ensuring allergy expertise for the future 

The potential of modern allergology can only be realized if the necessary expertise is sustainably available. Allergic diseases must be reliably diagnosed and treated in accordance with guidelines across the various areas of medical care; for severe and complex conditions, specialized allergological expertise is also required. 

This requires allergology expertise to be integrated into medical school curricula, as well as qualified continuing medical education and training. At the same time, allergology needs the next generation of scientists who can bridge clinical questions with basic and translational research. 

Ensuring allergological expertise is therefore a prerequisite for quality of care, patient safety, and scientific innovation. 

The five future tasks mark a fundamental shift: Allergology must evolve from a predominantly reactive approach to a more preventive, evidence-based, precision-oriented, and coordinated approach to medicine. To ensure that this medical potential reaches patients, the necessary structures must be created and sustainably secured. 

## 5. Structures for a sustainable future in allergology 

Allergology can only realize its full potential if research, training, and patient care are structurally interconnected. Allergology in 2030 therefore requires not isolated, individual measures, but a coordinated system: ranging from prevention and community-based care to specialized interdisciplinary expertise, university medicine, and translational research. 

Four structural areas are of central importance in this regard. 

### 5.1 Structurally anchoring university-based allergology and translational research 

An effective field of allergology requires visible and permanently established academic structures. University allergology must combine basic research, clinical research, teaching, specialized patient care, and the promotion of early-career researchers. 

This requires independent professorships in allergology and scientific research groups, as well as stronger networking among existing research centers. Interdisciplinary research consortia and clinical trial networks must facilitate the translation of new findings into diagnostics, prevention, and therapy, while simultaneously feeding questions arising from clinical practice back into research. 

University-based allergology is thus not merely a research center, but a central hub for innovation, training, and highly specialized care. 

### 5.2 Ensuring mandatory allergy expertise 

Allergy expertise must be reliably taught and maintained at all relevant levels of medical training. In medical school, fundamental knowledge of the prevention, diagnosis, treatment, and emergency management of allergic diseases should be firmly integrated in accordance with their epidemiological and clinical significance. 

Postgraduate medical training must ensure sufficient practical experience with the full spectrum of allergic diseases, diagnostic procedures, and therapies. In doing so, organ-specific specialist expertise and interdisciplinary allergological competence must be integrated. Structured training opportunities at qualified allergology centers can make a significant contribution to this [12, 13, 14]. 

Given the rapid pace of scientific advancement, continuous education in allergy remains indispensable. Clinical qualifications, support for early-career researchers, and attractive career paths must be considered together to ensure long-term expertise in allergy. 

### 5.3 Establishing a tiered and networked allergy care system 

A sustainable field of allergology requires a care continuum with different levels of allergological expertise. Common allergic diseases must be diagnosed and treated by qualified professionals close to where patients live. For severe, complex, and multimorbid conditions, specialized and interdisciplinary expertise must be accessible. 

To achieve this, outpatient allergy care, relevant medical specialties, specialized centers, and university medical centers must be linked through defined interfaces and care pathways. Patients should have access to the appropriate level of care based on the complexity of their condition and, following specialized diagnostics or therapy, should be able to continue receiving care as close to home as possible. 

CACs can serve as hubs for complex and severe conditions within such a system. In addition to providing interdisciplinary patient care, they can, in particular, take on roles in continuing education and training, clinical research, and translation, and contribute to the networking of allergy expertise [14, 16]. 

Prevention and early detection must be integral parts of this care continuum. In particular, the interfaces with pediatrics and primary care offer the opportunity to identify risk factors and early manifestations of disease, implement preventive measures, and, when necessary, initiate qualified allergy diagnostics at an early stage. 

### 5.4 Enabling a “learning” allergology through data, registries, and implementation 

Scientific evidence improves care only when it is implemented in clinical practice and its impact is evaluated. Allergy care in 2030 therefore requires structures that integrate research, guidelines, care, and care data into a continuous learning process. 

Registers, clinical trial networks, and health services research can track disease progression and treatment outcomes, identify gaps in care, and support the further development of guidelines and care models. Existing allergy registries and networks serve as important starting points for this [22]. 

Digital healthcare data has the potential to expand these possibilities. This requires interoperable and high-quality data structures, as well as high standards for data protection and scientific validity. Digital applications and AI-based methods should also be used where they offer demonstrable benefits for prevention, diagnosis, treatment, or follow-up care [23]. 

At the same time, a learning healthcare system must incorporate the perspective of those affected. High-quality patient information, education, and support for self-management are essential components of safe and effective care for chronic and potentially life-threatening allergic diseases. 

**From a structural vision to policy action**

The necessary building blocks of a sustainable allergy care system have thus been identified: strong university-based structures, assured expertise in allergology, a tiered and networked care system, and robust structures for translation, data, and implementation. 

Many of these elements already exist in Germany, but their availability varies by region; some are project-based or insufficiently interconnected. Existing expertise must therefore be strengthened, relevant gaps must be closed, and the individual elements must be integrated into a functioning overall system. 

This goes beyond the purview of individual professional societies, medical disciplines, and levels of care. The structural vision thus becomes a health policy mandate. 

## 6. From vision to policy action: A national action plan for allergology 

Germany possesses the scientific knowledge and medical capabilities to detect allergic diseases earlier, prevent them more effectively, and treat them better. The key challenge lies in sustainably establishing the necessary conditions in research, training, and care. 

The shortcomings in allergology are not limited to a single specialty, level of care, or institution. Accordingly, they cannot be resolved through isolated, individual measures. 

### Allergology is a shared responsibility in health policy 

From the DGAKI’s perspective, this gives rise to four overarching policy priorities: 

Ensure the structural and financial framework for guideline-based allergological care: Qualified, locally accessible care and specialized, interdisciplinary care for severe and complex diseases require framework conditions that enable guideline-based diagnosis and treatment, as well as the necessary collaboration between levels of care. 

Sustainably securing allergology expertise and academic structures: The burden of allergic diseases must be appropriately reflected in medical education, continuing medical education and training, as well as in university structures and scientific career paths. 

Structurally promote prevention, research, and translation: Evidence-based prevention and early detection, as well as scientific innovation, must be integrated into care more quickly and systematically. To achieve this, research, clinical trials, health services research, and appropriate data structures must be strengthened over the long term. 

Coordinate the further development of allergology at the national level: Prevention, care, training, research, and translation involve different stakeholders and areas of responsibility. They therefore require shared priorities and a binding framework for implementation and evaluation. 

The crucial political task, therefore, is not to simply list additional individual measures side by side, but to bring the necessary changes together into a unified strategy. 

### Germany Needs a National Action Plan for Allergology 

Over the past decades, Germany has repeatedly developed initiatives to improve allergy prevention and care. Nevertheless, there is still no comprehensive, long-term national framework that integrates prevention, care, training, research, and monitoring into a unified implementation and evaluation structure [24, 25, 26, 27]. 

The DGAKI therefore calls for the development of a National Action Plan for Allergology in Germany. Such an action plan must not be merely another catalog of desirable measures. It must be based on a systematic analysis of the disease burden and existing structural and care-related deficits, determine priorities in collaboration with relevant stakeholders, and translate these into concrete and measurable goals [27]. 

From the outset, its development must address the question of how prioritized goals can be implemented, responsibilities defined, necessary resources secured, and progress monitored. The action plan should utilize and integrate existing guidelines, networks, expertise, and healthcare structures. New structures are required where relevant gaps cannot be closed with existing tools [27]. 

A National Action Plan for Allergology is neither a substitute for medical guidelines nor the responsibility of a single scientific medical society for allergology and clinical immunology. Its added value lies in integrating scientific evidence, shared priorities, implementation, and evaluation into a long-term national strategy [27]. 

Its development requires the participation of policymakers, healthcare self-governance organizations, the scientific community, medical societies and associations, healthcare professionals, payers, and patient advocacy groups. The prioritization and operationalization of specific measures, responsibilities, and measurable goals must take place within a structured, collaborative process [27]. 

75 years after the founding of the DGAKI, it is time to derive a shared health policy mission from scientific progress and the accumulated expertise in allergology. Germany needs a national strategy so that allergic diseases can be detected earlier, prevented more effectively, and treated better in the future. The National Action Plan for Allergology is intended to create a shared framework for this. 

## 7. The DGAKI: Responsibility for the future of allergology 

For 75 years, the DGAKI has stood for the scientific and interdisciplinary advancement of allergology in Germany. It brings together medical specialties and scientific disciplines, thereby creating a platform for exchange between basic research, clinical research, and patient care [28]. 

This responsibility extends beyond the evaluation and advancement of scientific evidence. As a scientific and interdisciplinary medical society, the DGAKI helps to network allergology research, develop guidelines, promote young scientists and clinicians, support continuing education and training, and translate new findings into clinical practice [28]. 

### The future of allergology can only be shaped through collaboration 

The challenges described in this position paper extend beyond the purview of individual specialties, institutions, and organizations. A sustainable future for allergology therefore requires the collaboration of all relevant stakeholders. 

In this regard, the DGAKI expressly seeks close collaboration with other professional societies and associations active in allergology, in particular with the Society for Pediatric Allergology and Environmental Medicine (GPAU) and the Association of German Allergists (AeDA). Equally necessary is the involvement of patient advocacy groups, university medical centers, other medical societies and health professions, healthcare self-governance organizations, payers, and policymakers. 

Different perspectives and areas of responsibility are not a weakness, but rather a prerequisite for sustainable solutions. It is crucial to align them with common goals for prevention, research, training, and care. 

### 75 Years of DGAKI – Responsibility for the future 

With “Allergology 2030,” the DGAKI is deliberately linking its 75^th^ anniversary with a look toward the future. The scientific and medical possibilities for detecting allergic diseases earlier, preventing them more effectively, and treating them better have expanded considerably in recent years. The task now is to adapt the structural and health policy conditions accordingly. 

As a scientific medical society for allergology and clinical immunology, the DGAKI intends to take the initiative for a National Action Plan for Allergology and to shape its development scientifically in collaboration with its partners. 

The DGAKI is ready to contribute its scientific expertise, its interdisciplinary structure, and its networks to this collaborative process. 

The DGAKI’s 75^th^ anniversary is thus not only an occasion for reflection but also a mandate for the future: to detect allergic diseases earlier, prevent them more effectively, and treat them better – and to jointly create the scientific, structural, and political conditions for a sustainable future of allergology in Germany. 

## Acknowledgments 

The authors would like to thank the DGAKI office, Ms. Rudnik, and Ms. Zeyen for their editorial support. 

## Authors’ contributions 

E.H. developed the concept and initiated the drafting of the position paper. All authors contributed to the conceptualization of the content, the expert evaluation, and the drafting of the first manuscript version; E.H. prepared the final manuscript version. All authors read, revised, and approved the final version for publication. 

## Funding 

There was no external funding or support from commercial entities. 

## Conflict of interest 

Following disclosure, all authors unanimously declare that there is no conflict of interest with regard to this position paper. 
